# Identification of Spontaneous Shoulder Hemarthrosis with Point-of-Care Ultrasound in the Emergency Department

**DOI:** 10.5811/cpcem.2021.7.53408

**Published:** 2022-03-07

**Authors:** Kevin A. Padrez, William Shyy, Kavita Gandhi, Nancy Anaya, R. Starr Knight

**Affiliations:** University of California, San Francisco, Department of Emergency Medicine, San Francisco, California

**Keywords:** ultrasound, hemophilia, hemarthrosis

## Abstract

**Case presentation:**

A 32-year-old man with a history of hemophilia A presented to the emergency department with right shoulder pain, swelling, and decreased range of motion.

**Discussion:**

Emergency physicians can use ultrasound to quickly and accurately identify hemarthrosis at the bedside.

## CASE PRESENTATION

**Image 1:**
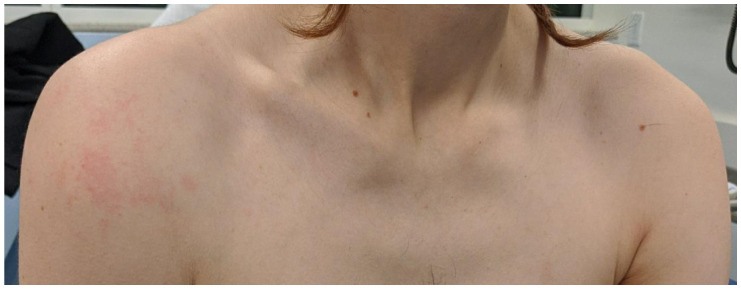
Photo of patient demonstrating asymmetric swelling of the right shoulder.

A 32-year-old man with a history of hemophilia A presented to the emergency department (ED) with right shoulder pain, swelling, and decreased range of motion. The patient had previously been treated with factor VIII replacement every three days for maintenance therapy but currently lacked access to established outpatient care after relocating from a nearby city. He was experiencing homelessness and sleeping outside. He denied preceding trauma, numbness, or weakness. His vital signs included: temperature 36.6ºC, heart rate 93 beats per minute, respiration rate 16 breaths per minute, blood pressure 173/127 millimeters mercury, and blood oxygen saturation of 100% on room air. His physical examination was remarkable for right shoulder swelling and tenderness along the right deltoid with decreased range of motion secondary to pain (Image 1). His distal motor and sensory function were intact.

The treating emergency physician performed a point-of-care ultrasound to evaluate for a right shoulder hemarthrosis. This exam was performed using a curvilinear 5–2 megahertz probe (Sonosite, Bothell, WA). While standing behind the patient, the probe was placed parallel to the ground just below the scapular spine. The probe marker was oriented to the patient’s left, which allows the anatomy on the screen to match the patient’s anatomy. The probe was then moved laterally until the glenoid, humeral head, and infraspinatus tendon were visualized. Both the affected and unaffected shoulders were evaluated using a similar technique.

**Image 2:**
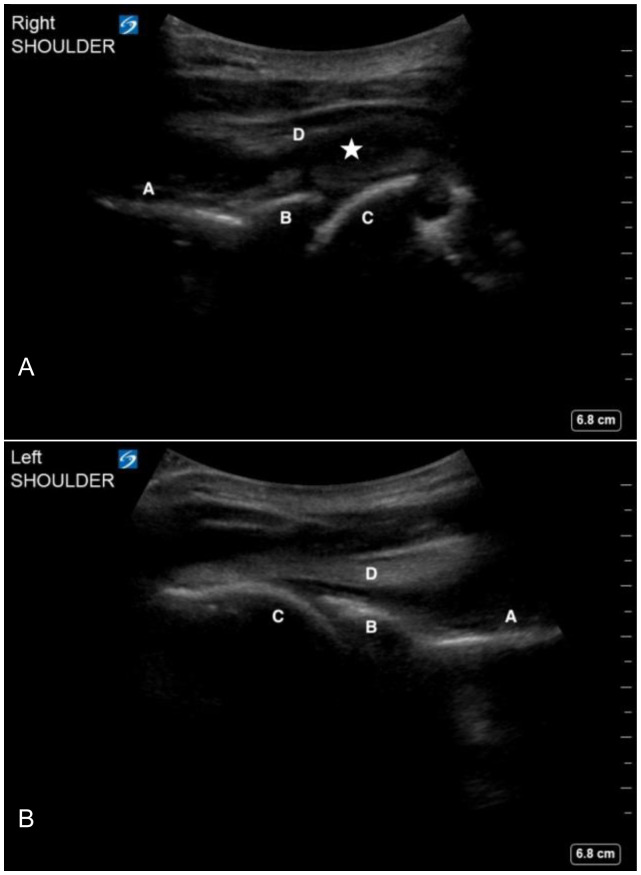
Ultrasound images of the affected right shoulder (2A) and unaffected left shoulder (2B) showing scapular spine (A), glenoid (B), humeral head (C) and infraspinatus tendon (D). A mixed-echotexture collection consistent with an intraarticular hemarthrosis (star) is visualized in the right shoulder.

In this patient, a collection with heterogeneous echogenicity was visualized above the humeral head and below the infraspinatus tendon, asymmetric to the unaffected side. These findings were consistent with a spontaneous hemarthrosis (Image 2). He was admitted to the hospital. Hematology was consulted and the patient was restarted on factor VIII treatment with improvement in his shoulder symptoms. He established care with the local hematology clinic to initiate regular factor VIII maintenance therapy.

## DISCUSSION

Spontaneous hemarthrosis is a common cause of morbidity and pain for patients with congenital hemophilia.^1^ While magnetic resonance imaging (MRI) is considered the conventional imaging modality for evaluating hemophilic arthropathy, ultrasound offers many benefits including less time, lower cost, and better accessibility than MRI.^2^ The detection of a joint effusion remains a core use of point-of-care ultrasound in the ED^3^ and has also been shown to accurately diagnose hemarthrosis in the outpatient setting.^4^,^5^ Emergency physicians can use point-of-care ultrasound to quickly and accurately identify hemarthrosis and expedite care for hemophilia patients.

The described technique to identify spontaneous hemarthrosis in the shoulder uses a curvilinear probe. The larger footprint of the probe allows for better visualization of the complete shoulder anatomy compared to a linear probe. However, a similar technique may be applied using the linear probe, especially in pediatric patients. Gentle internal and external rotation of the arm can help confirm the identification of the humeral head (video). Some small effusions may only be visible with external rotation of the affected arm.

**CPC-EM Capsule**

What do we already know about this clinical entity?

*Point-of-care ultrasound is commonly used to identify joint effusions*.

What is the major impact of the image(s)?

*Spontaneous shoulder hemarthrosis may be quickly and accurately identified using point-of-care ultrasound*.

How might this improve emergency medicine practice?

*Point-of-care ultrasound should be considered for the identification of spontaneous hemarthrosis in hemophilia patients presenting with joint pain*.

## Supplementary Information

Video Gentle internal and external rotation of the shoulder can help identify humeral head. Annotation identifies the glenoid (Gl), humeral head (HH), and hemarthrosis (star).
